# Maternal One-Carbon Metabolism and Infant DNA Methylation between Contrasting Seasonal Environments: A Case Study from The Gambia

**DOI:** 10.1093/cdn/nzy082

**Published:** 2018-10-12

**Authors:** Philip T James, Paula Dominguez-Salas, Branwen J Hennig, Sophie E Moore, Andrew M Prentice, Matt J Silver

**Affiliations:** 1 Medical Research Council Unit The Gambia at the London School of Hygiene and Tropical Medicine, London, United Kingdom; 2 Department of Production and Population Health, Royal Veterinary College, London, United Kingdom; 3 Population Health, Science Division, Wellcome Trust, London, United Kingdom; 4 Department of Women and Children's Health, King's College London, London, United Kingdom

**Keywords:** Developmental Origins of Health and Disease, DNA methylation, maternal nutritional status, metastable epialleles, nutritional epigenetics, one-carbon metabolism, seasonality

## Abstract

**Background:**

The periconceptional period is a time in which environmentally induced changes to the epigenome could have significant consequences for offspring health. Metastable epialleles (MEs) are genomic loci demonstrating interindividual variation in DNA methylation with intraindividual crosstissue correlation, suggesting that methylation states are established in the very early embryo before gastrulation. In our previous Gambian studies, we have shown that ME methylation states in the offspring are predicted by maternal concentrations of certain nutritional biomarkers around the time of conception.

**Objective:**

We aimed to assess whether the profile of maternal biomarker predictors of offspring methylation differs between rainy and dry seasons in a population of rural Gambians, using a larger set of 50 recently identified MEs.

**Methods:**

We measured 1-carbon biomarkers in maternal plasma back-extrapolated to conception, and cytosine-phosphate-guanine (CpG) methylation at 50 ME loci in their infants’ blood at a mean age of 3.3 mo (*n* = 120 mother-child pairs). We tested for interactions between seasonality and effects of biomarker concentrations on mean ME methylation *z* score. We used backward stepwise linear regression to select the profile of nutritional predictors of methylation in each season and repeated this analysis with biomarker principal components (PCs) to capture biomarker covariation.

**Results:**

We found preliminary evidence of seasonal differences in biomarker-methylation associations for folate, choline, and homocysteine (interaction *P* values ≤0.03). Furthermore, in stratified analyses, biomarker predictors of methylation changed between seasons. In the dry season, vitamin B-2 and methionine were positive predictors. In the rainy season, however, choline and vitamin B-6 were positive predictors, and folate and vitamin B-12 were negative predictors. PC1 captured covariation in the folate metabolism cycle and predicted methylation in dry season conceptions. PC2 represented the betaine remethylation pathway and predicted rainy season methylation.

**Conclusions:**

Underlying nutritional status may modify the association between nutritional biomarkers and methylation, and should be considered in future studies.

## Introduction

The Developmental Origins of Health and Disease (DOHaD) hypothesis suggests that early-life environmental exposures affect lifelong health and disease risk (1–4). For example, exposure to the Dutch Hunger Winter famine in 1944–1945 across different stages of prepregnancy and pregnancy has been associated with lower birthweight (5), increased adult blood pressure and obesity (6–8), and increased risk of schizophrenia (9). One plausible mechanism for these associations is through epigenetic modifications to the genome (10–12). Epigenetic processes encompass mitotically heritable changes to the genome that can alter gene expression without changing the underlying DNA sequence (13), and include DNA methylation (predominantly at cytosine-phosphate-guanine (CpG) sites), histone modifications, and RNA-based mechanisms (14).

Times of increased cell turnover, such as during fetal development, may be particularly susceptible to epigenetic errors or to adaptive modifications designed to capture early environmental cues (15, 16). Early embryonic development is a period of complex epigenetic remodeling and cell differentiation (17–19), and thus represents a critical window in which changes to the epigenetic program could have significant consequences for offspring health (20).

Metastable epialleles (MEs) are genomic loci whose (nongenetically determined) methylation state varies between individuals, but in whom variation is correlated across tissues originating from all 3 germ layers in a single individual (16, 21). This suggests the establishment of stochastic methylation states in the first few days after conception before separation into germ layers around gastrulation. ME methylation therefore provides a useful measure for studying the potential influence of the periconceptional environment on selected regions of the offspring epigenome (22, 23). ME methylation status in humans has been associated with obesity, immune function, and certain cancers (24–26).

A variety of nutritional and other environmental factors can impact the infant epigenome in utero through maternal exposure (20, 27, 28), including 1-carbon metabolites in the periconceptional period and during embryonic development (29). One-carbon metabolism refers to the interlinking reactions of the folate, choline, methionine, homocysteine, transsulfuration and transmethylation metabolic pathways (30, 31). DNA methylation is one of the numerous transmethylation reactions made possible by the donation of a methyl group from *S*-adenosylmethionine (SAM), forming *S*-adenosyl homocysteine (SAH) in the process (32). The SAM:SAH ratio has therefore been used as a proxy indicator of methylation potential (33). The 1-carbon pathways that enable transmethylation to occur rely on nutritional inputs in the form of methyl donors (e.g., folate, choline, betaine) and essential cofactors (e.g., vitamins B-2, B-6, and B-12) (30, 34). Nutritional status of the mother can therefore influence DNA methylation, and this is most clearly exemplified in animal models. In Agouti mouse experiments, pregnant dams fed a diet rich in vitamin B-12, folic acid, choline, and betaine gave birth to pups exhibiting increased methylation at the locus influencing the expression of the *Agouti* gene compared with controls. This resulted in changes to offspring fur color, appetite, adiposity, and glucose tolerance (27, 35). In humans, there is also evidence linking maternal nutrition to offspring DNA methylation, explored either as individual micronutrients or as proxy measures of nutrition such as famine and seasonality (36, 37). Although there is also much evidence linking DNA methylation to later phenotype (12, 38), studies fully exploring the continuum of maternal nutrient exposure, offspring DNA methylation, and later phenotype are relatively rare (39).

In a series of studies in rural Gambia, we have been able to exploit a seasonal “natural experiment,” whereby a cycling pattern of rainy and dry seasons imposes strikingly different environmental, especially nutritional, exposures on the population. We have shown that plasma collected in nonpregnant women of child-bearing age contains higher concentrations of methyl donors and has a higher methylation potential in the peak rainy season (July to September) than in the peak dry season (February to April) (40). Furthermore, we found that seasonal differences in maternal periconceptional nutritional status are associated with offspring methylation at multiple MEs. Increased concentrations of vitamin B-2 and decreased concentrations of vitamin B-6, homocysteine, and cysteine predicted increased offspring mean methylation across 6 MEs (23), whereas offspring conceived in the rainy season had consistently higher level of ME methylation in peripheral blood monocytes than those conceived in the dry season (22–26). However, our previous analyses did not explicitly test for an interaction with season for the associations between biomarker predictors and methylation.

Here, by exploring nutrient-season interactions, we extended our previous analyses to investigate whether the profile of maternal nutritional predictors of ME methylation varies between rainy and dry seasons. In doing so, we used a recently identified larger set of MEs associated with Gambian season of conception (SoC)-associated MEs (26) and explored in greater detail how covariation in the nutritional biomarkers can be captured in a principal components (PCs) model.

## Methods

This paper utilizes data from 2 parallel studies: the Methyl Donors and Epigenetics (MDEG) study (23) and the Early Nutrition & Immune Development (ENID) Trial (41), both conducted in the rural West Kiang region of The Gambia.

### Study population: The MDEG study

The MDEG study investigated the effects of periconceptional maternal biomarkers on infant DNA methylation at 6 candidate MEs (23). Women of reproductive age (18–45 y) were invited to participate and were followed monthly until pregnancy confirmation. Consenting women who conceived in the peak of the rainy season (July to September 2009) and the peak of the dry season (February to April 2010) were enrolled. Women provided a 10-mL fasting venous blood sample at the point they reported their first missed menses [mean (SD) 8.6 ± 4 weeks of gestation]. The following maternal 1-carbon biomarkers were analyzed: plasma folate, vitamin B-12, active vitamin B-12, choline, betaine, dimethylglycine (DMG), methionine, SAM, SAH, homocysteine (Hcy), cysteine, 4-pyridoxic acid (PA), pyridoxal (PL), pyridoxal-5ʹ-phosphate (PLP), and erythrocyte riboflavin (vitamin B-2), as described previously (23). All biomarkers were back-extrapolated to the time of conception using seasonal trends from a cohort of 30 nonpregnant women from the same district, who provided fasted venous blood samples every month for a year, as previously detailed (40). Infant DNA was obtained from a 3-mL venepuncture taken 2–8 mo after delivery. In this analysis, we use a subset of 120 infants for whom we had analyzed genome-wide DNA methylation data (Gene Expression Omnibus accession GSE59592), obtained using the Illumina Infinium HumanMethytlation450 array (“450k array”) (25, 42).

### Selection of season of conception-associated ME loci from the ENID study

ME loci were identified using data from the ENID trial. Participants in ENID partially overlap with those in the MDEG study, although in the analysis described here, individuals from MDEG and ENID form distinct, nonoverlapping groups. *n* = 50 SoC-associated ME loci were identified as the intersection between loci identified in a recent screen for MEs on the 450k array and 2171 CpGs showing SoC-associated differential methylation using 450k data from 128 ENID blood samples from infants aged 24 mo (26). Selection of loci demonstrating both metastability and sensitivity to the periconceptional environment, each in independent samples, strengthens evidence that they are established in the early embryo (16, 25, 26). We provide details on the locations and genomic context of the 50 CpGs used in this analysis in **Supplementary Table 1.** Our use of ENID samples to identify SoC-associated MEs in this analysis carries a number of advantages. First, it offers an opportunity to validate observations of increased rainy SoC ME methylation across independent ENID and MDEG 450k methylation datasets. Second, annual patterns of Gambian seasonality mean that potential confounding due to the relation between SoC and season of sample collection is different between ENID (median age of collection 24 mo) and MDEG (median age 3 mo) cohorts, enabling more robust inference (43). Third, SoC effects identified using ENID infant 24 mo DNA are by definition more persistent than those identified in younger MDEG samples, making them potentially more robust candidates for use as biomarkers or mediators of later health outcomes.

### Statistical analyses

#### Outcome: infant DNA methylation at 50 CpGs

The 50 SoC-associated ME loci on the 450k array identified using ENID methylation data (see above) were carried forward for use as candidates in the current analysis with 450k methylation data from 120 infants in the MDEG dataset, for which we had matching maternal plasma biomarker concentration data.

DNA methylation β values were adjusted for batch effects (25). CpG methylation across the 50 ME loci was highly correlated (Cronbach's α test reliability coefficient of 0.908). We therefore derived a univariate measure of ME methylation by converting methylation at each CpG into a *z* score [(individual observation – CpG mean)/CpG SD] and taking the mean of the methylation *z* scores across all 50 CpGs as our primary outcome measure.

#### Exposure variables: nutritional biomarkers

After removing variables demonstrating colinearity, the final list of nutritional exposure variables was: folate, active vitamin B-12, vitamin B-2, choline, betaine, DMG, SAM:SAH, Hcy, methionine, PLP, and cysteine. All nutritional biomarkers were treated as continuous variables. All biomarkers were log-transformed to improve normality, apart from SAM:SAH, which was left untransformed, and standardized before analyses.

In order to capture covariation of the 1-carbon biomarkers, we also conducted a PC analysis. Four PCs had an eigenvalue >1 and underwent orthogonal varimax rotation. We generated individual PC scores based on these loadings and used the resulting 4 PC variables in subsequent regression analyses.

#### Baseline characteristics

Because this study uses a subsample of 120 mother-child pairs from the original sample (*n* = 166) (23), we report the baseline characteristics again by SoC. Means (for continuous, normal data) were compared using Student's *t* test; medians (of non-normal data) were compared using the Wilcoxon rank-sum test; and proportions were compared using a chi-squared test or Fisher exact test (for categories with sparse data).

#### Associations between nutritional exposures and infant DNA methylation: crude analyses

To validate the findings from the original MDEG study using this larger set of MEs, we ran linear regression models to assess the crude association between maternal nutritional exposures and SoC with infant mean methylation *z* score. To assess the hypothesis that the profile of nutritional predictors might change between seasons, we then included season as an interaction term in the association between the nutritional exposures and methylation, assessing the interaction using the likelihood ratio test.

#### Primary objective: Predictors of methylation by SoC

Given that the interaction tests justified stratifying the data by SoC, we then explored the predictors of methylation in each season separately using multivariable linear regression. We used an automatic backward stepwise approach for variable selection, using a *P* value of >0.2 as the criterion for removal from the model. Each regression model was run twice; first, using the individual 1-carbon biomarkers as exposures (“biomarker model”) and second using the 4 PCs model.

All regression model residuals were checked for normality and met the assumptions of linear regression models. All models included 11 a priori confounders: maternal age, BMI, and gestational age at time of sample collection; infant sex and infant age at time of sample collection; and 5 methylation-derived white blood cell counts (25). All of these have previously been associated with methylation (44–47). We report likelihood ratio test results comparing the full model against the baseline model including a priori confounders only. Stata 14.0 (Stata Corporation) was used for all statistical analyses.

### Ethical considerations

Ethical approvals for the ENID trial and MDEG study were given by The Gambia Government/MRC Joint Ethics Committee (SCC1126v2 and SCC1151, respectively). Consent was gained by signature or thumb print from mothers for their own participation and that of their child. All data were anonymized before analyses.

## Results

**FIGURE 1:**
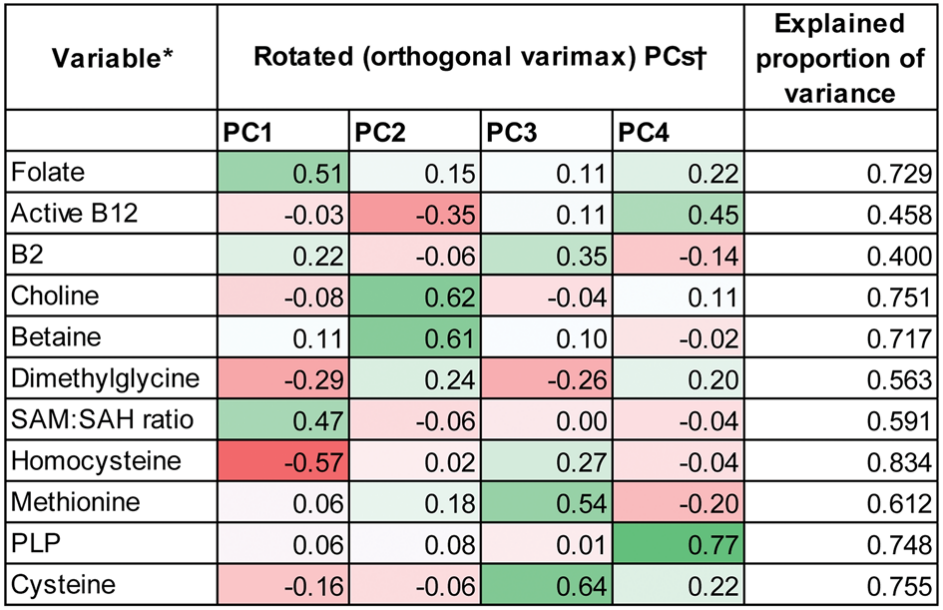
Heat map showing correlation between 1-carbon biomarkers and PCs after orthogonal rotation. ^*^All log-transformed, standardized variables, apart from SAM:SAH, which is standardized but untransformed. ^†^Cells shaded green for positive correlations and red for inverse correlations. Intensity of shading is proportional to strength of correlation. B12, vitamin B-12; B2, vitamin B-2; PC, principal component; PLP, pyridoxal-5ʹ-phosphate; SAH, *S*-adenosyl homocysteine; SAM, *S*-adenosyl methionine.

Four PCs with an eigenvalue >1 explained 65.0% of the total variation seen in the 11 biomarkers (**Supplementary Table 2**). After rotation, PC1 was associated positively with folate and SAM:SAH, and inversely with Hcy. PC2 was strongly correlated positively with choline and betaine, and inversely with active vitamin B-12. PC3 was positively correlated with the amino acids methionine and cysteine, and PC4 was strongly correlated with PLP and active vitamin B-12. These 4 PCs explained more than half the variability of all biomarkers apart from active vitamins B-12 and B-2, which still had 54.3% and 60.0% unexplained respectively. **Figure 1** shows the correlation between the 1-carbon biomarkers and the PC loadings as a heat map.

Baseline maternal and infant characteristics are summarized in **Table 1**, detailed for the overall sample and by SoC. There was no difference in maternal age, gestational age, maternal BMI, or infant sex by SoC. Infants conceived in the dry season were ∼0.5 mo younger at their DNA blood draw than were those conceived in the rainy season. Women conceiving in the rainy season had higher concentrations of folate, vitamin B-2, betaine, cysteine, and SAM:SAH, and lower concentrations of DMG, PLP, and Hcy than did those conceiving in the dry season. There were higher scores for PC1 and PC3, and lower scores for PC4 in the rainy season. We provide a detailed breakdown of the nutritional status of the population, stratified by season, in **Table 2**. Almost all women were deficient in vitamin B-2, >40% had low PLP status, ∼30% and 20% had low concentrations of betaine and choline respectively, 13% were folate-deficient, and participants were replete in methionine and cysteine. There was evidence to suggest that a greater proportion of women were folate-deficient in the dry season and that a higher proportion had low PLP concentrations in the rainy season.

**TABLE 1:** Maternal and infant characteristics, overall and stratified by season of conception^1^

	Total		Dry season		Rainy season		
Variable	*n*	Statistic	*n*	Statistic	*n*	Statistic	*P* ^2^
Folate, nmol/L	117	15.4 (14.3–16.6)	59	12.6 (11.6–13.7)	58	18.9 (17.0–20.9)	<0.001
Active B_12_, pmol/L	118	73.7 (68.1–79.8)	59	79.2 (71.4–87.8)	59	68.6 (60.7–77.6)	0.077
B_2_, 1/EGRAC	113	0.45 (0.43–0.48)	57	0.41 (0.38–0.44)	56	0.51 (0.47–0.55)	<0.001
Choline, µmol/L	117	6.6 (6.2–6.70)	59	6.9 (6.3–7.5)	58	6.3 (5.9–6.8)	0.109
Betaine, µmol/L	118	18.8 (17.6–20.1)	59	17.5 (15.7–19.4)	59	20.2 (18.6–21.9)	0.033
Dimethylglycine, µmol/L	118	2.2 (1.9–2.4)	59	2.9 (2.5–3.3)	59	1.6 (1.4–1.8)	<0.001
SAM:SAH ratio	118	7.7 (7.2–8.2)	59	6.5 (6.0–7.1)	59	9.1 (8.4–9.7)	<0.001
Homocysteine, µmol/L	118	6.7 (6.3–7.2)	59	7.4 (6.8–8.1)	59	6.1 (5.7–6.6)	<0.001
Methionine, µmol/L	118	24.4 (23.5–25.3)	59	22.9 (21.8–24.1)	59	26.0 (24.7–27.3)	<0.001
Pyridoxal phosphate, nmol/L	118	21.9 (20.2–23.7)	59	23.7 (20.9–27.0)	59	20.1 (18.4–22.1)	0.041
Cysteine, µmol/L	118	197.3 (192.9–201.9)	59	192.6 (186.8–198.5)	59	202.2 (195.6–209.0)	0.032
PC1	111	0.00 ± 1.58	57	−0.89 ± 1.24	54	0.94 ± 1.36	<0.001
PC2	111	0.00 ± 1.34	57	−0.03 ± 1.46	54	0.04 ± 1.21	0.788
PC3	111	0.00 ± 1.26	57	−0.52 ± 1.14	54	0.55 ± 1.15	<0.001
PC4	111	0.00 ± 1.13	57	0.35 ± 1.18	54	−0.37 ± 0.95	<0.001
Maternal age, y	117	29.2 ± 6.7	58	28.2 ± 6.1	59	30.3 ± 7.2	0.089
Gestational age, d	120	60.7 ± 28.5	60	63.8 ± 27.8	60	57.6 ± 28.9	0.237
BMI, kg/m^2^	120		60		60		0.866
Underweight		16.7 ± 20		15.0 ± 9		18.3 ± 11	
Normal		72.5 ± 87		73.3 ± 44		71.7 ± 43	
Overweight		10.8 ± 13		11.7 ± 7		10.0 ± 6	
Infant sex, % (*n*)	120		60		60		0.534
Female		48.3 (58)		51.7 (31)		45.0 (27)	
Male		51.7 (62)		48.3 (29)		55.0 (33)	
Infant age, mo	112	3.3 [3.11–3.77]	55	3.2 [3.07–3.21]	57	3.7 [3.31–4.0]	<0.001

1 Values are geometric means (95% CIs) for maternal biomarkers; medians [IQRs] for infant age; means ± SDs for PCs, maternal age, gestational age, and BMI; and % (*n*) for infant sex. B_12_, vitamin B-12; B_2_, vitamin B-2; EGRAC, erythrocyte glutathione reductase activity coefficient; PC, principal component; SAH, *S*-adenosyl homocysteine; SAM, *S*-adenosyl methionine.

2 Testing difference by season: Wilcoxon rank-sum test for nonnormal data, Student's *t*-test for normal data, chi-squared test for proportion.

**TABLE 2:** Maternal plasma biomarker status, overall and stratified by season of conception^1^

		Overall (both seasons)		Dry season		Rainy season		
Variables	Cutoff for low/abnormal status	*n* below cutoff	%	*n*	%	*n*	%	*P* ^2^
Homocysteine	>15 µmol/L (48)	2/118	1.7	2/59	3.4	0/59	0.0	0.496
Folate	<10 nmol/L (49)	15/117	12.8	12/59	20.3	3/58	5.2	0.024
B_2_	<0.77 (1/EGRAC) (50)	109/113	96.5	57/57	100.0	52/56	92.9	0.057
PLP	<20 nmol/L (51)^3^	50/118	42.4	19/59	32.2	31/59	52.5	0.025
Active B_12_	<37 pmol/L (52)	6/118	5.1	1/59	1.7	5/59	8.5	0.207
Choline	<5 µmol/L (48)^3^	24/117	20.5	12/59	20.3	12/58	20.7	0.963
Betaine	<16 µmol/L (48)^3^	35/118	29.7	22/59	37.3	13/59	22.0	0.070
Methionine	<20 µmol/L (53)^4^	19/118	16.1	12/59	20.3	7/59	11.9	0.210
Cysteine	<36 µmol/L (53)^4^	0/118	0.0	0/59	0.0	0/59	0.0	—

1 B_12_, vitamin B-12; B_2_, vitamin B-2; EGRAC, erythrocyte glutathione reductase activity coefficient; PLP, pyridoxal-5′-phosphate.

2 Test for seasonal difference in biomarker status. *P* values from chi-squared test, or Fisher's exact test (if any numerator <5).

3 There are no clearly defined plasma cutoffs for deficiency. The suggested cutoffs indicate below the normal range and can be considered “low status.”

4 The amino acid cutoffs represent the 10th percentile of a healthy population age >16 y in Canada (53). Note that these cutoffs do not necessarily represent low status or deficiency.

The crude association between mean total CpG methylation (*z* scores) and each exposure (SoC, nutritional biomarkers and PCs) is shown in **Table 3**. Total mean methylation across the 50 CpG sites was 0.26 *z* scores higher in the rainy season than in the dry season (95% CI: 0.07, 0.45; *P* = 0.008). The SAM:SAH ratio was positively associated with methylation, and there was weak evidence to suggest that Hcy was inversely associated. Among the PCs, only PC1 was positively associated with methylation.

**TABLE 3:** Crude association between exposures and total mean CpG methylation *z* score, overall and stratified by season using linear regression^1^

	Overall (both seasons)			Stratified analysis by season			
Variable^2^	*n*	Coefficient (95% CI)^3^	*P* ^4^	Season	Coefficient (95% CI)^3^	*P* ^4^	*P* interaction^5^
Season^6^	109	0.26 (0.07, 0.45)	0.008				
Log folate	108	0.02 (−0.07, 0.11)	0.623	Dry	0.10 (−0.06, 0.26)	0.237	0.019
				Rainy	−0.14 (−0.26, −0.01)	0.034	
Log active B_12_	109	−0.08 (−0.17, 0.02)	0.102	Dry	−0.04 (−0.18, 0.09)	0.549	0.758
				Rainy	−0.07 (−0.18, 0.05)	0.264	
Log B_2_	105	0.04 (−0.05, 0.14)	0.347	Dry	−0.01 (−0.16, 0.14)	0.916	0.986
				Rainy	−0.01 (−0.14, 0.13)	0.924	
Log choline	108	0.02 (−0.07, 0.11)	0.642	Dry	−0.02 (−0.13, 0.09)	0.705	0.030
				Rainy	0.16 (0.02, 0.3)	0.023	
Log betaine	109	0.05 (−0.04, 0.14)	0.236	Dry	0.02 (−0.09, 0.13)	0.661	0.661
				Rainy	0.06 (−0.08, 0.2)	0.397	
Log dimethylglycine	109	−0.03 (−0.12, 0.06)	0.496	Dry	−0.01 (−0.15, 0.12)	0.869	0.227
				Rainy	0.10 (−0.05, 0.26)	0.175	
SAM:SAH ratio	109	0.12 (0.03, 0.20)	0.010	Dry	0.16 (0.02, 0.31)	0.029	0.086
				Rainy	0.00 (−0.14, 0.14)	0.986	
Log homocysteine	109	−0.09 (−0.18, 0.00)	0.054	Dry	−0.13 (−0.25, −0.01)	0.039	0.030
				Rainy	0.06 (−0.08, 0.21)	0.389	
Log methionine	109	0.05 (−0.04, 0.15)	0.277	Dry	0.09 (−0.03, 0.22)	0.132	0.062
				Rainy	−0.07 (−0.21, 0.07)	0.341	
Log PLP	109	0.05 (−0.04, 0.15)	0.277	Dry	0.00 (−0.11, 0.11)	0.999	0.762
				Rainy	−0.03 (−0.18, 0.13)	0.727	
Log cysteine	109	−0.05 (−0.14, 0.04)	0.303	Dry	−0.06 (−0.19, 0.07)	0.352	0.955
				Rainy	−0.06 (−0.18, 0.07)	0.371	
PC1	103	0.06 (0.00, 0.12)	0.037	Dry	0.13 (0.03, 0.22)	0.015	0.002
				Rainy	−0.07 (−0.16, 0.02)	0.139	
PC2	103	0.04 (−0.03, 0.11)	0.299	Dry	0.01 (−0.07, 0.10)	0.763	0.255
				Rainy	0.09 (−0.02, 0.19)	0.123	
PC3	103	0.00 (−0.09, 0.08)	0.909	Dry	−0.02 (−0.13, 0.09)	0.762	0.399
				Rainy	−0.08 (−0.21, 0.04)	0.191	
PC4	103	−0.06 (−0.14, 0.02)	0.126	Dry	−0.03 (−0.13, 0.08)	0.628	0.837
				Rainy	−0.04 (−0.18, 0.09)	0.533	

1 B_12_, vitamin B-12; B_2_, vitamin B-2; CpG, cytosine-phosphate-guanine; PC, principal component; PLP, pyridoxal-5′-phosphate; SAH, *S*-adenosyl homocysteine; SAM, *S*-adenosyl methionine.

2 All 1-carbon biomarkers log-transformed and standardized, apart from SAM:SAH (standardized only).

3 Adjusted for maternal BMI at time of bled, gestational age at time of bleed, maternal age, infant sex, infant age, white blood cell composition.

4 Two-tailed *t*-test for coefficient slope.

5 Likelihood ratio test comparing models with and without interaction term.

6 Season is coded 0 = dry 1 = rainy.

To justify stratified analyses, we first tested whether there was any interaction between the effect of the exposure on total mean methylation by SoC (Table 3). There was some evidence of an interaction with SoC for plasma folate, choline, and homocysteine (*P* value for interaction = 0.019, 0.030, and 0.030, respectively). There was no evidence of an interaction with season for any of the other biomarkers, and overall effect sizes remained small. For the PCs, only PC1 showed a different pattern of association with methylation by season (*P* value for interaction = 0.002).

In stratified analyses using backward stepwise regression, Hcy, vitamin B-2, methionine, and SAM:SAH were retained in the dry season multivariable biomarker model (**Table 4**). Of these selected variables, methionine was the strongest positive predictor of methylation, followed by SAM:SAH. Hcy was associated with decreasing methylation as in crude analyses, as was vitamin B-2. The full model explained 27.0% of total variance in methylation (adjusted *R*^2^, model *P* = 0.001). In the dry season, PC model PC1 was the only covariate retained, and the model explained 18.7% of methylation variance (model *P* = 0.009).

**TABLE 4:** Multivariable linear regression: predictors of methylation (dry season)^1^

Biomarker model			PC model		
Variable^2^	Coefficient (95% CI)^3^	*P* ^4^	Variable	Coefficient (95% CI)^3^	*P* ^4^
Log homocysteine	−0.16 (−0.31, −0.01)	0.040	PC1	0.15 (0.03, 0.26)	0.012
Log methionine	0.17 (0.04, 0.30)	0.011			
Log B_2_	−0.10 (−0.25, 0.06)	0.214			
SAM:SAH ratio	0.13 (−0.05, 0.31)	0.164			
*n*	—	52			52
Overall model *P*^5^	—	0.001			0.009
*R*-squared	—	0.471			0.362
Adjusted *R*-squared	—	0.270			0.187

1 B_2_, vitamin B-2; PC, principal component; SAH, *S*-adenosyl homocysteine; SAM, *S*-adenosyl methionine.

2 All 1-carbon biomarkers log-transformed and standardized, apart from SAM:SAH (standardized only).

3 Adjusted for maternal BMI at time of bleed, gestational age at time of bleed, maternal age, infant sex, infant age, white blood cell composition.

4 Two-tailed *t* test for coefficient slope.

5 Likelihood ratio test comparing the final model with the model only including a priori confounders.

In the rainy season biomarker model, a different profile of predictors was retained. SAM:SAH, choline, and PLP were associated with increasing methylation, whereas folate and active vitamin B-12 were associated with decreasing methylation (**Table 5**). The rainy season model explained 9.4% of methylation variance (adjusted *R*^2^, model *P* = 0.004). In the rainy season PC model, PC2 was positively associated with methylation. PC1 and PC3 were also retained and showed weak inverse associations. The model, however, fitted poorly.

**TABLE 5:** Multivariable linear regression: predictors of methylation (rainy season)^1^

Biomarker model			PC model		
Variable^2^	Coefficient (95% CI)^3^	*P* ^4^	Variable	Coefficient (95% CI)^3^	*P* ^4^
Log folate	−0.20 (−0.35, −0.06)	0.008	PC1	−0.06 (−0.16, 0.03)	0.188
Log active B_12_	−0.08 (−0.20, 0.04)	0.201	PC2	0.09 (−0.01, 0.20)	0.080
Log PLP	0.11 (−0.08, 0.30)	0.236	PC3	−0.08 (−0.20, 0.04)	0.169
Log choline	0.18 (0.03, 0.32)	0.018			
SAM:SAH ratio	0.14 (−0.02, 0.30)	0.093			
*n*	—	51			51
Overall model *P*^5^	—	0.004			0.052
*R*-squared	—	0.366			0.243
Adjusted *R*-squared	—	0.094			−0.023

1 B_12_, vitamin B-12; PC, principal component; PLP, pyridoxal-5′-phosphate; SAH, *S*-adenosyl homocysteine; SAM, *S*-adenosyl methionine.

2 All 1-carbon biomarkers log-transformed and standardized, apart from SAM:SAH (standardized only).

3 Adjusted for maternal BMI at time of bleed, gestational age at time of bleed, maternal age, infant sex, infant age, white blood cell composition.

4 Two-tailed *t*-test for coefficient slope.

5 Likelihood ratio test comparing the final model with the model only including a priori confounders. PC, principal component.

**FIGURE 2:**
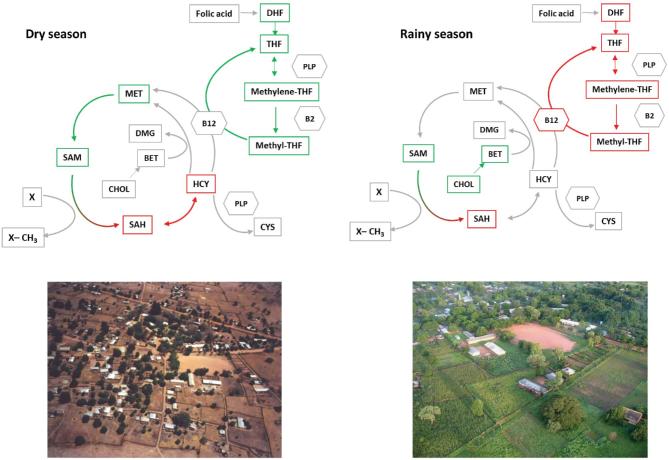
Simplified summaries of metabolic pathways acting as positive (green) and negative (red) predictors of methylation by season of conception, drawn from the retained principal-component coefficients in the multivariate models. Photos show Keneba, West Kiang district, The Gambia. Photo credit: Andrew Prentice. BET, betaine; B12, vitamin B-12; B2, vitamin B-2; CHOL, choline; CYS, cysteine; DHF, dihydrofolate; DMG, dimethyl glycine; Hcy, homocysteine; MET, methionine; methylene-THF, *N*^5,10^-methylene tetrahydrofolate; methyl-THF, *N*^5^-methyl tetrahydrofolate; PLP, pyridoxal-5ʹ-phosphate; SAH, *S*-adenosyl homocysteine; SAM, *S*-adenosyl methionine; THF, tetrahydrofolate; X, acceptor; X-CH_3_, methylated acceptor.

A graphical summary of the associations between predictors of methylation retained in the multivariable models by SoC is shown in **Figure 2**. This figure simplifies the above results by focusing on the PC associations, showing the switch of positive predictors of methylation from the folate pathway in the dry season to the choline/betaine pathway in the rainy season.

## Discussion

This study extends our understanding of previously reported associations between 1-carbon biomarkers in mothers at the time of conception and DNA methylation at MEs in their infants. We have validated previous findings of increased methylation at MEs for rainy season conceptions, but found that maternal plasma biomarkers back-extrapolated to the time of conception generally demonstrate little individual effect on infant ME methylation, whether in crude analyses or in multivariable predictive models. There was some preliminary evidence to suggest an interaction between SoC and the association of maternal 1-carbon biomarkers with infant methylation.

### PCs and metabolic pathways

The PC approach is useful for exploring covariation in biomarkers and their joint influence on methylation, although their biological interpretation can be difficult. However, our findings suggested the strongest loadings of each PC mapped onto different metabolic pathways. The major loadings for PC1 are involved in the folate metabolism cycle. The major form of folate in plasma is 5-methyl-tetrahydrofolate (54), which donates its methyl group to Hcy via methylene tetrahydrofolate reductase using vitamin vitamin B-12 as a coenzyme (55). The remethylation of Hcy forms methionine, which is then used to form SAM, thus explaining why the SAM:SAH ratio loading is also positively correlated with this PC along with folate. The inverse correlation of Hcy is expected because it is held in equilibrium with SAH, a buildup of which can impede the SAM to SAH reaction via product inhibition of methyltransferases (56). In contrast, PC2 loadings are positively associated with the betaine remethylation pathway. Choline is the precursor to betaine, which is formed via a 2-step oxidation reaction (57). Betaine donates its methyl group to homocysteine, catalyzed by betaine-homocysteine methyl transferase (57). Methionine and cysteine provide the major loadings for PC3. This could represent the transsulfuration pathway, because methionine provides the sulfur atom for cysteine synthesis, via the irreversible degradation of Hcy (31). It could also reflect that methionine and cysteine are dietary components found in similar food sources. The PC4 primary loading comes from PLP, which is particularly involved in 1-carbon metabolism as a coenzyme in the transsulfuration pathway, as well as being required to reduce THF to methylene-THF (31).

### Crude analyses between SoC, 1-carbon related exposures, and methylation

Using an expanded set of 50 ME CpGs associated with SoC in samples from older infants (26), we validated our previous finding (23) of increased ME methylation in rainy season conceptions in the younger cohort analyzed here. Biomarker concentrations differed by season in ways that have been previously described (23), forming a profile with higher methylation potential in the rainy season than in the dry season. In the original MDEG study, we found that periconceptional concentrations of vitamin B-2 were positively associated with offspring methylation and Hcy, whereas vitamin B-6 and cysteine were inversely associated (23). In these current analyses, we found the same association with Hcy, but not with vitamin B-2, vitamin B-6, or cysteine. Instead, in crude analyses, we found that SAM:SAH was positively associated with methylation, in line with the expected effect of these intermediary metabolites on methylation potential (33). The differences between the current and previous analyses could reflect the reduced sample size in this updated analysis (due to the smaller number of samples with Illumina 450k array data), additional adjustment covariates used, or the larger panel of MEs used to derive a univariate methylation score in the current study. The associations reported here help explain why there is evidence of a crude association between PC1 and methylation. Homocysteine has been associated with decreased methylation in several cross-sectional studies (58). Taken in isolation, folate did not show any association with methylation in this study, and this has also been the case in other studies (59–61). However, folate did load strongly onto PC1, which showed a positive association with methylation in the dry season model. Increased maternal periconceptional folate status has been associated with increased methylation in infants at a differentially methylated region of retinoid X receptor alpha (*RXRA*) (62). However, this pattern is not consistent, and inverse associations between fetal periconceptional folate exposure and methylation have also been found at syntaxin 11 (*STX11*), orthodenticle homeobox 2 (*OTX2*), transcription factor AP-2 alpha (*TFAP2A*), cystin 1 (*CYS1*), and leptin (*LEP*) (39, 62, 63)*.*

### Predictors of methylation by SoC from multivariable analyses

In the dry season, the predictors broadly indicate that increasing methylation potential (increasing SAM:SAH and decreasing Hcy, most likely through the folate pathway looking at the PC1 loadings) contributes to higher levels of DNA methylation. However, in the rainy season, when there is higher plasma folate and lower plasma Hcy than in the dry season, the folate pathway unexpectedly switches to an inverse association, and we can hypothesize that the betaine remethylation pathway takes prominence. Although these simple regression models cannot address the specific molecular mechanisms involved, we can speculate on a few different possibilities. In the rainy season, we could be seeing the effect of feedback loops attenuating the influence of increased plasma folate. One-carbon metabolism is governed by intricately controlled feedback loops, which help protect the flux of metabolites through key reactions over a range of nutrient and cofactor concentrations (64, 65). Alternatively, it could be that the rainy season folate metabolism is at saturation, and the system can then only enhance SAM:SAH through the betaine remethylation pathway. This highlights the complexity of 1-carbon metabolism in human populations and suggests that the potential for effect modifiers, for example season in our Gambian setting, should be considered when modeling methyl donor pathways. Given the exploratory nature of our analyses and the small effect sizes reported, our findings need to be replicated in confirmatory studies.

### Limitations

There are a number of unmeasured exposures that may follow a seasonal pattern and could contribute to differences in offspring methylation, thus confounding our results. These include other nutrition-related exposures [e.g., vitamin A (66), vitamin D (67, 68), and dietary polyphenols (69)], as well as other potential exposures such as maternal stress (70), toxin exposure (71), intrauterine growth restriction (72–74), maternal hyperglycemia (75), infection (76), and seasonal differences in the microbiome (77). Future nutritional intervention studies will help establish whether there is a causal association between differences in diet, nutritional biomarkers, and methylation.

Linear regression models rest on certain assumptions and have a number of limitations. For example, when modeling the effects of nutritional factors, minimum detection thresholds and saturation effects will introduce nonlinear effects that cannot be captured by linear regression. Stepwise regressions allow large numbers of predictors to be evaluated, but have been criticized for producing inflated coefficients, and for the fact that after the strongest predictor has been considered, there is little additional explanatory power for any correlated predictor (78). They are also known to be relatively unstable in that small changes in the data can cause one variable to be selected over another, which can then alter subsequent variable selection (79). It is therefore possible that some of the differences we see between the seasons are related to model instability rather than reflecting real changes. This point is exacerbated by the fact that our sample size was small, meaning stratified analyses by season may not have had adequate power to distinguish true differences.

The use of PCs gives some further insight into the joint effect of correlated biomarkers, offering an analysis strategy that lies conceptually between the consideration of biomarkers in isolation, and more sophisticated approaches that attempt to model the full complexity of metabolic networks. PC regression models are, however, hard to interpret. There are other models that are designed to help generate an understanding of how 1-carbon pathways interact (often in nonlinear ways), for example by estimating fluxes of metabolites through the pathways under given scenarios, and within specific cellular compartments (50, 80–82). Although these models are mathematically sophisticated, many are based on kinetic data that can be difficult to obtain at the population level. Furthermore, there is a need to generate models that can integrate plasma concentration data, the most common and accessible type of experimental data used for human in vivo studies. A promising way forward is within the field of systems biology, an integrative discipline that analyses complex datasets to help generate hypotheses, which can be experimentally validated and used to improve computer modeling in an iterative fashion (83). However, despite the limitations of the linear regression models we used, they can still play a role in hypothesis generation.

### Conclusions

Before this study, we had observed that methylation at 6 MEs is higher among infants conceived in the rainy season than in those conceived in the dry season, and this trend has been seen again in a larger set of 50 MEs in the current analysis. However, we had not previously investigated whether the same combination of methyl donors and cofactors were consistently associated with methylation, or whether there was an interaction with season. In this current analysis, we find preliminary evidence to suggest that the rainy and dry seasons in The Gambia have a different set of maternal nutritional predictors of infant methylation. However, larger sample sizes and more sophisticated ways of modeling the complex nonlinear interrelations of metabolites are needed to further our understanding of what might trigger a switch between different methylation pathways at the molecular level.

Although there is still much work to do to complete our understanding of underlying mechanisms, our findings highlight potential considerations for future study design. If underlying nutritional status (partially captured in this study by the observed seasonal variations in plasma biomarker concentrations) influences the predictors of DNA methylation, then this would be applicable to populations with heterogeneous patterns of dietary intake, whether seasonally driven or otherwise. This suggests that studies would benefit from collecting detailed information on nutritional status to assess if underlying nutritional status acts as an effect modifier. In observational studies, this information may help to explain contradicting associations between nutrition and other environmental exposures and DNA methylation or, in the case of trials, between nutritional interventions and DNA methylation. Such considerations might also inform the timing of future studies if there are seasonal dietary intake variations, or the targeting of subgroups in the context of populations with broad variation in nutritional status. In summary, the underlying nutritional status could be an essential piece of information to help disentangle the often complex and contradictory findings from nutritional epigenetics studies.

## Supplementary Material

Supplement Tables

## Notes

This work was supported by a Wellcome Trust grant WT086369MA (to BJH), core funding MC-A760-5QX00 to the International Nutrition Group by the UK Medical Research Council and the UK Department for the International Development under the MRC/DFID Concordat agreement, and by the MRC grant for the “Impact of maternal diet on the epigenome” (MC_EX_MR/M01424X/1).

The Gene Expression Omnibus accession number for the original 450k data sets is GSE59592.

Author disclosures: PTJ, PD-S, BJH, SEM, AMP, MJS, no conflicts of interest.

Supplemental Tables 1 and 2 are available from the “Supplementary data” link in the online posting of the article and from the same link in the online table of contents at https://academic.oup.com/cdn/.

Abbreviations used:

CpG: cytosine-phosphate-guanine
DMG: dimethylglycine
DOHaD: Developmental Origins of Health and Disease
ENID: Early Nutrition and Immune Development
Hcy: homocysteine
MDEG: Methyl Donors and Epigenetics
ME: metastable epiallele
PA: 4-pyridoxic acid
PC: principal components
PL: pyridoxal
PLP: pyridoxal-5ʹ-phosphate
SAH: *S*-adenosyl homocysteine
SAM: *S*-adenosyl methionine
SoC: season of conception
