# Supplementary material for: Maternal One-Carbon Metabolism and Infant DNA Methylation between Contrasting Seasonal Environments: A Case Study from The Gambia
Source: Curr Dev Nutr. 2018 Oct 12;3(1):nzy082. doi: 10.1093/cdn/nzy082 (PMC6351729; doi:10.1093/cdn/nzy082)
Supplement: Supplement Tables [file nzy082_supplement_file.docx]

**Supplementary Table 1: Location and genomic context of the 50 CpGs used in analyses***

| **CpG ID** | **Chromo-some** | **Position** | **Gene name (UCSC)** | **Gene region feature category (UCSC)** | **Relationship to Canonical CpG Island**^†^ | **Enhancer element** |
| --- | --- | --- | --- | --- | --- | --- |
| cg08787039 | 1 | 182921857 | C1orf14 | Body | Island |  |
| cg15025200 | 1 | 152161237 |  |  | North Shore |  |
| cg18388270 | 1 | 149212246 |  |  |  |  |
| cg17598339 | 2 | 170624727 |  |  | Island | Yes |
| cg00817731 | 3 | 46759475 | PRSS50 | TSS200 | Island |  |
| cg01788113 | 3 | 46759472 | PRSS50 | TSS200 | Island |  |
| cg02834909 | 3 | 46759438 | PRSS50 | TSS200 | Island |  |
| cg16975614 | 3 | 105601834 |  |  |  |  |
| cg19668234 | 3 | 46759449 | PRSS50 | TSS200 | Island |  |
| cg20814179 | 4 | 940893 | TMEM175 | 5'UTR | Island |  |
| cg23206463 | 5 | 561769 |  |  | North Shore |  |
| cg02735058 | 6 | 16181250 |  |  |  |  |
| cg09894276 | 6 | 169977394 | WDR27 | Body | Island |  |
| cg10218546 | 6 | 32729823 | HLA-DQB2 | Body | Island |  |
| cg15189031 | 6 | 34499314 | PACSIN1 | Body | Island |  |
| cg18609891 | 6 | 32729647 | HLA-DQB2 | Body | Island |  |
| cg23414861 | 6 | 74064064 | DPPA5 | TSS200 | Island |  |
| cg08767820 | 7 | 51539583 |  |  |  | Yes |
| cg10614809 | 7 | 51539190 |  |  |  | Yes |
| cg14329783 | 7 | 75779857 |  |  | Island |  |
| cg06565641 | 8 | 141359674 | TRAPPC9;TRAPPC9 | Body;Body | South Shore | Yes |
| cg07092985 | 8 | 55533887 | RP1 | Body | Island |  |
| cg07234876 | 8 | 600039 |  |  | Island |  |
| cg23958373 | 8 | 599963 |  |  | Island |  |
| cg06159404 | 10 | 43846376 |  |  | Island |  |
| cg01794156 | 11 | 397077 | PKP3 | Body | Island |  |
| cg05940452 | 11 | 118842484 | FOXR1;FOXR1 | 5'UTR;1stExon | Island |  |
| cg23188684 | 11 | 67383651 |  |  | Island | Yes |
| cg11258452 | 12 | 119594454 | SRRM4 | Body | South Shelf |  |
| cg23887609 | 12 | 130822674 | PIWIL1;PIWIL1 | 1stExon;5'UTR | Island |  |
| cg24229701 | 12 | 130821962 | PIWIL1 | TSS1500 | North Shore |  |
| cg24838063 | 12 | 130822603 | PIWIL1 | TSS200 | Island |  |
| cg26677194 | 12 | 130822605 | PIWIL1 | TSS200 | Island |  |
| cg14089267 | 13 | 23412409 |  |  | Island |  |
| cg14395298 | 13 | 23412250 |  |  | Island |  |
| cg25576711 | 14 | 24780557 | LTB4R2;LTB4R2;LTB4R2CIDEB;CIDEB;LTB4R | Body;1stExon;Body;  1stExon;5'UTR;TSS200 | Island |  |
| cg26310551 | 14 | 24780540 | LTB4R2;LTB4R2;LTB4R2CIDEB;CIDEB;LTB4R | Body;1stExon;Body;  1stExon;5'UTR;TSS200 | Island |  |
| cg01232511 | 16 | 2867446 | PRSS21;PRSS21;  PRSS21 | Body;Body;Body | Island |  |
| cg08288433 | 17 | 20799511 | CCDC144NL | TSS200 | Island |  |
| cg13590055 | 18 | 77917647 | LOC100130522;LOC100130522;PARD6G | Body;Body;3'UTR | Island |  |
| cg21237861 | 18 | 77918142 | PARD6G;LOC100130522;LOC100130522 | Body;Body;Body | Island |  |
| cg26211724 | 18 | 57636670 |  |  | Island |  |
| cg02452966 | 19 | 39056216 | RYR1;RYR1 | Body;Body | Island |  |
| cg05345154 | 19 | 2250901 | AMH | Body | Island |  |
| cg15035382 | 19 | 40728255 | CNTD2 | 3'UTR | Island |  |
| cg17434634 | 19 | 49522954 |  |  | Island | Yes |
| cg19605788 | 19 | 40729386 | CNTD2 | Body | Island | Yes |
| cg19712189 | 19 | 33210851 | TDRD12;TDRD12 | 5'UTR;1stExon | Island |  |
| cg24923931 | 19 | 40904691 | PRX;PRX | Body;Body | Island |  |
| cg13403462 | 20 | 32256071 | NECAB3;NECAB3;C20orf134;C20orf134 | Body;Body;1stExon;3'UTR | South Shore |  |

*****All details from Infinium HumanMethylation450K Manifest File v1.2 using coordinates from Genome Build 37.

^†^Shores - 0-2 kb from CpG island; Shelves - 2-4 kb from CpG island.

**Supplementary Table 2: Principal components explaining variation in 11 biomarkers of 1-carbon metabolism**

| **Principal Component (PC)** | **Eigenvalue** | **Proportion of variance explained** | **Cumulative proportion of variance explained** |
| --- | --- | --- | --- |
| PC1 | 2.70 | 0.25 | 0.25 |
| PC2 | 1.99 | 0.18 | 0.43 |
| PC3 | 1.40 | 0.13 | 0.55 |
| PC4 | 1.06 | 0.10 | 0.65 |
| PC5 | 0.93 | 0.08 | 0.74 |
| PC6 | 0.78 | 0.07 | 0.81 |
| PC7 | 0.73 | 0.07 | 0.87 |
| PC8 | 0.47 | 0.04 | 0.92 |
| PC9 | 0.40 | 0.04 | 0.95 |
| PC10 | 0.33 | 0.03 | 0.98 |
| PC11 | 0.21 | 0.02 | 1.00 |
